# Prevalence and Risk Factors of Myopia in Young Adults: Review of Findings From the Raine Study

**DOI:** 10.3389/fpubh.2022.861044

**Published:** 2022-04-27

**Authors:** Samantha Sze-Yee Lee, David A. Mackey

**Affiliations:** ^1^Centre for Ophthalmology and Visual Science (Incorporating the Lions Eye Institute), University of Western Australia, Perth, WA, Australia; ^2^Centre for Eye Research Australia, Royal Victorian Eye and Ear Hospital, University of Melbourne, East Melbourne, VIC, Australia; ^3^School of Medicine, Menzies Research Institute Tasmania, University of Tasmania, Hobart, TAS, Australia

**Keywords:** axial length, the Raine Study, myopia, risk factors, young adults, education, sun exposure, time spent outdoors

## Abstract

Myopia tends to develop and progress fastest during childhood, and the age of stabilization has been reported to be 15–16 years old. Thus, most studies on myopia have centered on children. Data on the refractive error profile in young adulthood — a time in life when myopia is thought to have stabilized and refractive error is unaffected by age-related pathology such as cataract — are limited. The Raine Study has been following a community-based cohort of young adults representative of the general Western Australia population since their prenatal periods in 1989–1991, with eye examinations performed when participants were 20 and 28 years old. At 20 years old, prevalence of myopia in the cohort was 25.8%. Using long-term trajectory of serum vitamin D levels and conjunctival ultraviolet autofluorescence (CUVAF) area to objectively quantify sun exposure, the Raine Study confirmed a negative relationship between time spent outdoors and myopia prevalence. However, prospective studies are required to determine the amount of CUVAF area or serum vitamin D levels associated with time duration. Combining data from the Raine Study and several other cohorts, Mendelian randomization studies have confirmed a link between myopia and a genetic predisposition toward higher education. Several novel potential associations of myopia or ocular biometry were investigated, including fetal growth trajectory, which was found to be significantly associated with corneal curvature at 20 years. By age 28, myopia prevalence had increased to 33.2%. Between 20 and 28 years old, myopia progressed and axial length elongated, on average, by −0.041D/year and 0.02 mm/year, respectively. Smaller CUVAF area at follow-up, female sex, and parental myopia were significant risk factors for myopia incidence and progression between 20 and 28 years. Given the limited research in young adults, further investigations are warranted to confirm the Raine Study findings, as well as identify novel genetic or environmental factors of myopia incidence and progression in this age group.

## Introduction

Eye conditions tend to arise during childhood or older adulthood, thus most studies of eye diseases and refractive error have involved children or older adults. Conversely, the eye health of young adults has received limited attention in the literature (1). Young adulthood tends to be a period when eye health and vision is expected to be at its peak, and when refractive error, especially myopia, has stabilized while unaffected by cataracts. Studying young adults, rather than measuring ocular parameters during childhood when eye development is still occurring, may inform on early life and childhood factors that may influence eye health. The eye examinations in the Raine Study aimed to address this gap in the literature. This article summarizes the myopia findings arising from the Raine Study, with a focus on the risk factors and progression of myopia during young adulthood.

## The Raine Study

In 1989–1991, 2,900 pregnant women were recruited from obstetric clinics in Perth, Western Australia (2). The study explored the effect of frequent ultrasound scans during pregnancy on birth outcomes and formed a cohort for studying the effects of early life events on later health outcomes. The women were randomly assigned to an “intensive imaging” group (ultrasound and Doppler imaging performed at 18, 24, 28, 34, and 38 weeks' gestation) or the control group (standard single ultrasound scan at 18 weeks') (3). Children born to these women (n= 2,868) formed the original study cohort (Gen2) and have been undergoing a series of various health and medical examinations from birth. With the enrolment of their parents (Gen1), grandparents (Gen0), and children (Gen3), the Raine Study has become one of the longest running multigenerational cohort studies in the world.

A main strength of exploring associations with myopia or other refractive error in the Raine Study Gen2 cohort is that it is generally representative of the general Western Australia adult population of similar age (4). At birth, and at the 8-, 14-, 17-, 20-, and 22-year follow-ups, elements of participants' socioeconomic profile, such as employment and income levels, were all within 7% difference from that of the West Australian population. However, because Gen2 participants were recruited before birth, an inherent limitation of the Raine Study is that the majority are Caucasian (~85.5%) and all were born in the state, as opposed to the rest of the state which has seen the number of overseas-born residents increase from 32.2% in 2001 to 39.7% in 2016. Additionally, a gradual loss to follow-up due to the longitudinal nature of the study has occurred, but participants and non-participants of the 20-year follow-up had similar birth and demographic characteristics (4). The representativeness of the Gen2 sample at the 28-year follow-up requires evaluation, although its profile is not expected to be markedly divergent from that of the general population.

### Eye Examinations

The Gen2 20-year follow-up was conducted from March-2010 to February-2012, when 1,344 participants (46.9% of the original cohort of 2,868) had their first eye examination as part of the study. Data collected from this allowed us to document the prevalence of refractive error, pterygium, and keratoconus (5–7), and profile the normative optical coherence tomography-derived parameters (8–11), in community-based young adults.

The Gen2 28-year follow-up eye examination (12) was conducted from March-2018 to March-2020 and attended by 813 participants (28.3% of original cohort; it is worth noting that data collection for this follow-up ended early because of the COVID-19 pandemic). This follow-up documented the longitudinal change in eye measures in young adults, with a focus on the 8-year change in refraction and optic disc measures. Both follow-ups included ocular biometry and cycloplegic autorefraction using the same instrument models (IOLMaster V.5; Carl Zeiss Meditec AG and Nidek ARK-510A; NIDEK, respectively).

## Myopia at 20 Years Old

Based on the International Myopia Institute definitions (13), 25.8% of the Raine Study participants had myopia at age 20 (spherical equivalent [SphE] ≤-0.50 D in either eye) (14), including 5.5% with SphE ≤-3.00 D and 1.5% with ≤-6.00 D (high myopia) (6, 14).

### Confirmation of Risk Factors by the Raine Study

#### Time Spent Outdoors

Many studies quantified time spent outdoors using self- or parent-reported data, which is subject to recall bias, especially if the data were collected retrospectively (15). Light sensors such as actigraphs can quantify time spent outdoors objectively; however, participants are required to wear the device on a regular basis and this approach is typically only used for short-term data collection.

Our eye provides natural markers of sun exposure: the presence of pterygia and the amount of conjunctival ultraviolet autofluorescence (CUVAF). While pterygium is uncommon in young adults, areas of CUVAF are measurable in adults and children, although it is less common in younger children (16). Like pterygium, the formation of CUVAF is due to the Coroneo effect, where light rays enter at an oblique angle, through the cornea and crystalline lens, and focus at the limbal area. With more UV entering from the temporal aspects, the light rays are focused at the nasal bulbar conjunctiva. Thus, CUVAF and pterygium tend to be larger and more common at the nasal than the temporal bulbar conjunctiva (17). Just as actinic damage on the skin fluoresces under short wavelength light due to cellular changes from chronic sun exposure (18), actinic changes in the bulbar conjunctiva secondary to UV exposure cause affected areas to fluoresce under low-level UV light, which can be photographed and measured using specialized instruments.

Using CUVAF to quantify sun exposure, the Raine Study confirmed a significant relationship between sun exposure and myopia. Myopia rates in the participants in the lowest quartile of CUVAF area (indicating less sun exposure) were more than double those in the highest quartile (33 vs. 16%) (19). Total CUVAF area (right+left eyes) was also significantly smaller in those with myopia compared to non-myopes (median= 31.9 vs. 47.9 mm^2^) (19). The authors pointed out that this difference in CUVAF area was unlikely to be related to the use of spectacles or contact lenses, as demonstrated by the similar CUVAF area in myopes who did and did not normally wear optical correction (31.9 vs. 31.6 mm^2^) and hyperopes who did and did not wear optical correction (43.8 vs. 49.1 mm^2^). When only the participants who wore optical correction were included, a significant difference in CUVAF area was still found between myopes and hyperopes (31.9 vs. 43.8 mm^2^). Even though spectacles often now have UV-filters, these may not provide protection against UV rays entering the eyes at oblique angles, which are responsible for CUVAF formation.

Serum vitamin D is an objective measure of recent time spent outdoors. In concordance with previous observations of the link between less time spent outdoors and myopia, the Raine Study found an inverse relationship between serum vitamin D levels and myopia, after correcting for sex, ethnicity, parental myopia, and CUVAF area (20, 21), with an odds ratio (OR) of 0.88 for myopia for every 10 nmol/L increase in vitamin D levels at age 20. Statistical significance was found for vitamin D measured in recent years (17- and 20-year) but not during childhood (6-year) or adolescence (14-year) (21). This finding appears at odds with the notion that sun exposure during early childhood may be protective against myopia. The authors suggest that the lack of statistical significance could be because vitamin D may be a poorer indicator of sun exposure at younger ages, although it is not clear why this may be the case and this has yet to be verified (The limitations of using serum vitamin D as a marker for time outdoors are discussed in the next sub-section). The authors also suggested that insufficient study power, where there were fewer participants with vitamin D measurements at 6 and 14 years (*n* = 618 and 988, respectively), could also explain why no relationship between these variables was found (21).

##### Strengths and Limitations of Objective Measures of Time Outdoors

Using CUVAF area and serum vitamin D levels to quantify sun exposure has significant advantages: the measures are objective and do not require participants to use any special device (e.g., actigraphs). CUVAF area is unaffected by dry eyes (22) and measures long-term sun exposure (23, 24), which may be more relevant for the study of myopia development and progression than short-term sun exposure measures. Vitamin D levels can provide information on short-term sun exposure.

However, both methods are more difficult to obtain compared to self-reported data. CUVAF photography requires specialized camera lenses and electronic filtered flash, then measurement of the CUVAF area manually or by an automated program (22, 24, 25). Even though manual measurement of CUVAF area is subjective, the intra- and interobserver reliability of CUVAF area measurements is high (correlation coefficients of both >0.9) (26). While CUVAF is generally a good representation of cumulative long-term sun exposure, shrinking of CUVAF area with age has been observed (24, 27), possibly because of use of sunglasses during adulthood and development of cataracts (thus allowing less UV to enter the eye) in older age. CUVAF may therefore become less accurate as a measure of sun exposure with older age. Serum vitamin D analysis requires collecting blood samples, which may be considered too invasive for some people, especially children. Another drawback of these markers is that a time duration is not quantified, i.e., how much time spent outdoors, as quantified using CUVAF, is associated with a unit decrease in myopia risk or progression. Prospective studies should be undertaken to explore this. The use of sunglasses (which tends to provide more coverage against UV light entering from oblique angles than conventional prescription glasses) or UV-blocking contact lenses, may influence the area of CUVAF (19, 24), while use of sunscreen can reduce synthesis of vitamin D. Actual time spent outdoors would then be underestimated using these approaches (24, 28).

#### Education

Several studies have confirmed that higher education is a risk factor for myopia (29, 30). Fan et al. (30) performed a meta-analysis of the gene–environment interaction effect, combining data from the Raine Study together with results from 33 other studies totaling over 50,000 participants. Participants who completed education beyond secondary school were, on average, 0.59D more myopic than those who had not, with a greater impact of education in Asians compared to Caucasians (difference of −1.09D and −0.49D, respectively).

While Mendelian randomization studies have shown that a genetic predisposition to higher education is linked with higher risks of myopia (31, 32), we should be cautious in concluding a causal link between education and myopia as this relationship is likely confounded by other risk factors such as less time spent outdoors and increased near work (30).

##### Novel Data: Effects of Taking a Gap Year Between High School and University

With increased push to pursue tertiary and higher education, more individuals are likely to enroll in university. As myopia can start to develop and continue progressing in early adulthood (14), this may further drive the myopia epidemic. However, it is not prudent to discourage higher education as it contributes to individual wellbeing, economic growth, and advancements in science and technology. Given that myopia progression slows with age, taking a break from formal education during the late teenage years between high school and university may help to reduce overall myopia progression or risk of myopia onset, relative to completing all formal education early in life, through the teenage years when myopia may still progress quickly. We tested this hypothesis by exploring the association between taking a gap year after high school and myopia.

Of the 1,344 participants who attended the Gen2 20-year eye examination, 816 had refraction data and information on any gap years taken after high school. We did not find a significant difference in myopia prevalence between those who took a gap year and those who did not (26.3 vs. 23.5%, *p* = 0.70 adjusted for sex, ethnicity, CUVAF area, and eventual attainment of undergraduate degree). Similarly, there was no difference in SphE or axial length (AL). While participants who spent their gap year working had slightly longer eyes than those who spent it traveling (Estimate = 0.21 mm; 95% confidence interval [CI] = −0.01 to +0.43), this failed to reach statistical significance (*p* = 0.06).

Taking a gap year is a common experience among Australian high school graduates. The COVID-19 pandemic has reduced international travel and casual employment, resulting in many young people choosing to start their tertiary education immediately rather than taking a gap year (33). Our data suggest that skipping a gap year will not have a major impact on myopia progression or prevalence.

#### Birth Order

There has been some evidence that first-born children are at higher risk of myopia than later-born children. However, previous studies had defined myopia based on level of unaided vision (6/12 or worse) (34, 35). To address this, four cohorts were analyzed: Raine Study Gen2 (*n* = 1,344), Avon Longitudinal Study of Parents And Children (ALSPAC; *n* = 4,401), Singapore Cohort Study Of Risk factors of Myopia (SCORM; *n* = 1,959), and Israeli Defense Force Pre-recruitment Candidates (IDFP; *n* = 88,277) (36). The larger cohorts found significantly higher rates of myopia in first-born compared to later-born children (ALSPAC: OR = 1.31, 95%CI = 1.05–1.64; IDFP: OR = 1.04, 95%CI = 1.03–1.06). In the IDFP, the difference in myopia prevalence between first- and fourth-born children was larger than the difference between the first- and second- or third-born children. The associations between birth order and myopia rates were unlikely to be due to chance, given that the two smaller cohorts also found a trend, albeit without statistical significance (Raine: OR = 1.18, 95%CI = 0.90–1.55; SCORM: OR = 1.25, 95%CI = 0.89–1.77). This association was significant even after excluding “only children” (who are, by definition, first-born), suggesting that this link is not mediated by environmental risk factors after birth. Guggenheim et al. (37) further confirmed this association in the United Kingdom Biobank (first- vs. second-born children OR = 1.12; 95%CI = 1.08-1.16) and noted that this association was weakened when highest level of education was accounted for, suggesting that the link could be partly mediated by increased educational pressure on first-born children. However, given the small increase in odds of myopia in first-born children, the association between birth order and myopia is unlikely to be clinically significant.

### Explorations for Novel Risk Factors

The myriad of health and medical data collected by the Raine Study allowed us to explore for other potential risk factors of myopia that may otherwise be overlooked. In particular, information on gestation and birth parameters, activity and eating habits during childhood and adolescence collected prospectively can be used to identify early life associations of myopia.

### Fetal Growth

The human eye starts to develop in the first trimester of gestation (38). Thus, disruptions or alterations to this ocular developmental process may affect visual outcomes. Indeed, lower birth weight has been linked with steeper corneas and shorter AL (39, 40). Thus, myopia associated with low birth weight is pathophysiologically different from school-myopia, which tends to result from axial elongation.

Birth weight is frequently used as a measure of intrauterine growth, and neonates with low birth weight are often assumed to have intrauterine growth restriction. However, many neonates with low birth weight may be constitutionally small, e.g. because the mother has a small stature, and have no other evidence of fetal growth restriction or associated complications (41, 42). Using multiple ultrasound images taken during gestation is a better way to examine fetal growth. Approximately half of the original Raine Study cohort underwent an “intensive imaging” protocol during gestation (3), providing a unique opportunity to model longitudinal fetal growth for each participant.

Multiple ultrasound scans and refractive information were available for 498 Raine Study Gen2 participants. The ultrasound scans were used to model the fetal growth trajectory based on fetal anthropomorphic measures, including head circumference, abdominal circumference, femur length, and estimated fetal weight (43). Dyer et al. (43) found that participants with consistently short or consistently long femur length during gestation tended to have a higher prevalence of myopia (27–29%) at 20 years old compared to those who had moderate femur lengths during late gestation (i.e., those with medium, big, or accelerated growths; 14–22%, *p* = 0.04). This suggests that there may be some *in utero* factors at play in late gestation that disrupted the coordination between ocular biometric measures (43).

Additionally, steeper corneas were found in participants who had slower growth in head circumference, femur length, and estimated fetal weight. While shorter AL was noted in those with slower fetal growths, this association did not reach statistical significance, which could be related to the large environmental influences on AL by the time an individual reaches 20 years of age.

### Non-significant Risk Factors

As critical as it is to find risk factors for myopia, it is equally important to rule out other causal links and report these non-significant risk factors. Findings from the Raine Study have suggested limited associations of myopia (and other ocular parameters) with *in utero* ultrasound exposure (44), anesthesia exposure during childhood (45), sleep quality trajectory from childhood to adolescence (46), and dietary vitamin A intake (47). These are discussed briefly in the Supplementary Notes.

## Myopia Development and Progression During Young Adulthood

Reports have suggested that myopia tends to stabilize around 15–16 years old (48, 49). However, longitudinal studies in university students in their early 20's (50–54) have demonstrated that myopia can progress or even begin after adolescence. However, beyond these university (50–54) or myopia (48, 49) cohorts, there are limited data on myopia development and progression in young adults, especially in the general population. This gives the impression that myopia progression during young adulthood is related to the pursuit of higher education. With the rising proportion of the population in indoor-based occupations (55), even if no higher education is completed, myopia development and progression in young adults may not be confined to university students.

### Findings From the Raine Study

**Figure 1:**
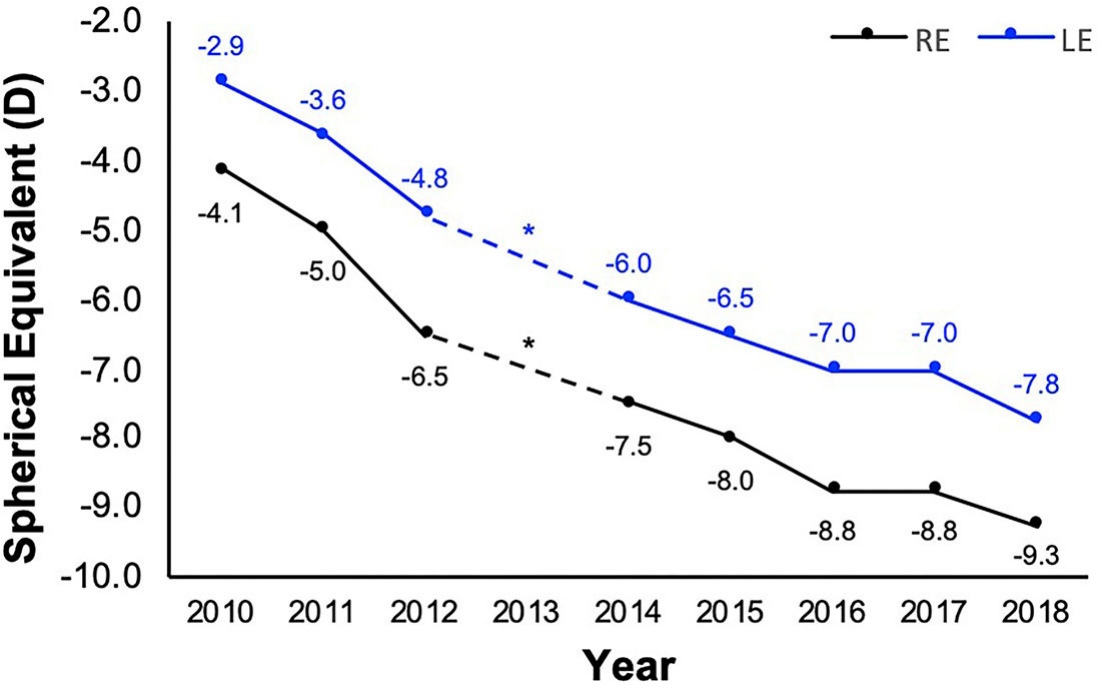
Refraction of a participant with rapid myopia progression from 20 (year 2010) to 28 years old (year 2018). Refraction (spherical equivalent) history obtained from participant's optometrist *apart from 2013 when the participant did not visit the optometrist. The participant reported their occupation during the 8-year period to be computer-intensive (at or close to 100%), with the exception of the year 2013 and mid-2016 to 2017, when most of their time was spent on outdoor academic field work or traveling. While no refraction data was available at 2013 (*), from 2016 to 2017, no myopia progression was documented.

Based on the data collected at the 20- (baseline) and 28-year Raine Study Gen2 follow-ups, the prevalence of myopia and high myopia increased from 25.8 to 33.2% and 1.4 to 1.5%, respectively, with incidence of 14% and 0.7% (35). While the majority (52.2%) of participants had a stable refraction in both eyes over the 8 years, about one-third (*n* = 261) of participants had a myopic shift (change in 0.50D over 8 years) in at least one eye, including 152 who progressed in both eyes. A novel case study is presented in Figure 1, demonstrating rapid myopia progression in one participant (~5D over 8 years).

Across all participants, SphE progression, axial elongation, and lens thickening were also observed over the 8 years, at average rates of −0.041D/year, 0.02 mm/year, and 0.220 mm/year, respectively (all *p* < 0.001), although corneal curvature did not change over time (14). Based on these findings, it appears that myopia progression in young adults has a similar mechanism to that in children, i.e., driven by axial elongation. This suggests that childhood risk factors of myopia, such as decreased time spent outdoors, may also have a myopigenic role during young adulthood. The sub-section below discusses these potential risk factors.

As shown in Table 1, the Raine Study cohort had lower annual myopia incidence and progression that those previously reported in young adults (50–54). This is most likely because previous studies included only university students, who may be spending less time outdoors due to their studies.

**Table 1:** Myopia incidence and progression in young adults, as reported by previous studies.

**References**	**Cohort**	**Follow-up duration**	**Myopia incidence**	**Myopia progression**
The Raine Study (*n* = 701)	Community-based; baseline age = 20 years	8 years	1.75%/year	−0.041 D/year
Jacobsen et al. (52) (*n* = 143)	Medical students; baseline age = 23 years	2 years	4.8%/year	−0.12 D/year
Jorge et al. (50) (*n* = 118)	University students; baseline age = 21	3 years	6.5%/year	−0.10 D/year
Jiang et al. (53) (*n* = 64)	Optometry students; baseline age = 25 years	9 months during school term	-	−0.37 D/year
Loman et al. (54) (*n* = 117)	Law students; age = 27 years	3 years, retrospective^a^	6.3%/year^a^	-
Kinge and Midelfart, (51) (*n* = 224)	Engineering students; baseline age = 21 years	3 years	11%/year^b^	−0.17 D/year

a *Based on participant-reported information*;

b *Kinge and Middelfart defines myopia as spherical equivalent ≤-0.25D*.

#### Risk Factors

In our study (14), we further tested the hypothesis that major risk factors of childhood myopia — parental myopia, less time spent outdoors, and higher education — are also associated with myopia progression in early adulthood. Indeed, for each parent with myopia, odds of incident myopia increased by 1.6 times, while SphE and AL progression rates were increased by 0.01D/year and 0.005 mm/year. Interestingly, level of education was not associated with myopia incidence or progression. While less time spent outdoors, as quantified by CUVAF area, was associated with incident myopia, it was not associated with myopia progression, as has been suggested in some studies, although findings on the latter observation have been conflicting (56–58).

We additionally found that women had higher odds of incident myopia (OR = 1.8) and double the progression rate compared to men (SphE and AL progression: women: −0.04D/year and 0.02 mm/year vs. men: −0.02D/year and 0.01 mm/year), after correcting for education and CUVAF area (14). Longitudinal studies in children have similarly noted that girls' myopia progressed faster than boys' (59–61), attributing this difference to pubertal growth spurts (62). However, this is unlikely to explain the sex difference seen in our cohort of young adults. Given that potential confounding factors of education and time spent outdoors have been accounted for in this study, this difference in myopia status between males and females could be influenced by other factors, such as ocular changes during pregnancy, which should be explored in future studies (63–65).

##### Novel Data: Sleep/Wake Time

Possible links exist between sleep parameters and myopia (66–70) (also see Supplementary Notes), although findings have been conflicting. Some cross-sectional studies (71, 72) have noted that myopes tend to go to sleep and rise later than non-myopes. In a 2-year longitudinal study of over 6,000 children, Liu et al. (73) similarly found a significant link between sleep/wake times and myopia. Children who went to sleep at 9:30 p.m. or later had a 1.45-OR for incident myopia and faster myopia progression by −0.16D, compared to those who went to sleep before 9 p.m. However, the 4-year longitudinal study by Wei et al. (74) failed to find such an association.

Here, we explored the relationship between sleep/wake time and myopia progression between 20- and 28-years in the Raine Study Gen2 cohort. At the 22-year follow-up (75), participants completed a questionnaire on their typical sleep and wake times on weekdays and weekends, and whether they considered themselves to be more of a “morning” or an “evening person.” A total of 620 participants had sleep/wake time information at the 22-year follow-up and refractive data at both the 20- and 28-year follow-ups. Linear mixed-effect models were used to explore the effect of sleep measures on longitudinal change in myopia measures, accounting for known confounders (CUVAF area, sex, ethnicity, and parental myopia) (14).

There was no obvious association between sleep time and SphE change. However, later times of falling asleep on weekends was associated with faster axial elongation by 0.003 mm/year for each hour delay in sleep time (95%CI = 0.000 to 0.004). A similar association was found for sleep time on weekdays but this was not significant (Estimate = 0.001 mm/year, 95%CI = −0.002 to 0.003).

Additionally, each hour delay in wake time on weekends was associated with increased rates of SphE and AL change by 0.006D/year (95%CI = 0.001 to 0.011) and 0.003 mm/year (95%CI = 0.001 to 0.005), respectively. A similar association was found for wake time during weekdays although this failed to reach statistical significance (SphE: Estimate = 0.001D/year, 95%CI = −0.003 to 0.005; AL: Estimate = 0.001 mm/year, 95%CI = −0.000 to 0.003). Sleep duration and self-report of “morning” or “evening” were not associated with myopia progression.

These findings along with those from other cross-sectional studies (71–73) suggest that falling asleep later was associated with a higher risk of myopia progression, even after accounting for sun exposure. While the effect of sleep and wake times found in the current analysis on young adults is small and unlikely to be clinically significant, it is possible that sleep/wake times may be more important in children when myopia progresses faster. The mechanism underlying this link is unclear, although Liu et al. (73) suggested that late-night near-work activities, such as reading, could confound this relationship. Future studies exploring sleep time or circadian rhythm and myopia should account for near work at night, for example, reading and using smart mobile devices in bed, to rule out any possible confounding effect of late-night near work. A disruption to the circadian rhythm with later time of falling asleep has also been suggested to be myopigenic, as the choroidal thickness and AL vary diurnally (76, 77). Genetic factors could also be at play, with Hysi et al. (78) recently reporting shared genes between refractive error and circadian rhythm.

## Conclusion and Future Directions

Findings from the Raine Study has confirmed many of the previously reported childhood risk factors of myopia and found fetal growth, and ruled out several other variables (*in utero* ultrasound exposure, childhood anesthesia exposure, sleep quality trajectory, dietary vitamin A), as a risk factor. Importantly, the Raine Study confirmed that myopia can begin or continue to progress in the third decade of life, and this change is not limited to those who studied at university. While refractive changes in young adulthood are generally smaller than those observed during childhood, we highlight that some individuals may still be susceptible to myopia progression at alarming rates. Further explorations are warranted to identify young adults who have rapid myopia progression. Given that myopia progression in young adults seems to have a similar mechanism and risk factors to those in children, it is worth investigating if myopia control intervention (e.g., pharmacological or optical interventions, or spending more time outdoors) may be beneficial in susceptible young adults. The differential rate of myopia progression between sexes also requires further investigation to understand the mechanism underlying this effect. Finally, it is critical that longitudinal birth cohort studies in other populations increase their focus on young adults given the historical lack of attention in this age group. The ALSPAC (79) and the Generation R cohort (80) are on track to accomplish this.

## Author Contributions

SL wrote the first draft of the manuscript and performed the statistical analysis. DM performed a supervisory role and acquired funded. All authors contributed to manuscript revision, read, and approved the submitted version.

## Funding

The core management of the Raine Study is funded by the University of Western Australia, Curtin University, Telethon Kids Institute, Women and Infants Research Foundation, Edith Cowan University, Murdoch University, the University of Notre Dame Australia and the Raine Medical Research Foundation. The eye examination for the Raine Study Gen2 20- and 28-year follow-ups were funded by the NHMRC (1021105, 1121979, and 1126494), the Heart Foundation (102170), Canadian Institutes of Health Research, Australian Foundation for the Prevention of Blindness, Alcon Research Institute, Telethon Kids Institute, Ophthalmic Research Institute of Australia, and the Lions Eye Institute (Perth, Australia). The authors would like to thank the National Health and Medical Research Council (NHMRC) for their long-term contribution to funding the study over the last 30 years. DM was supported by a NHMRC practitioner Fellowship.

## Conflict of Interest

The authors declare that the research was conducted in the absence of any commercial or financial relationships that could be construed as a potential conflict of interest.

## Publisher's Note

All claims expressed in this article are solely those of the authors and do not necessarily represent those of their affiliated organizations, or those of the publisher, the editors and the reviewers. Any product that may be evaluated in this article, or claim that may be made by its manufacturer, is not guaranteed or endorsed by the publisher.
