# Supplementary material for: Prevalence and Risk Factors of Myopia in Young Adults: Review of Findings From the Raine Study
Source: Front Public Health. 2022 Apr 27;10:861044. doi: 10.3389/fpubh.2022.861044 (PMC9092372; doi:10.3389/fpubh.2022.861044)
Supplement: Supplementary file 1 [file Data_Sheet_1.DOCX]

**Supplementary note: unlikely associations of myopia**

### *In utero* ultrasound exposure

The Hawthorne effect, whereby the act of measurement itself changes the outcome (in this case, performing multiple ultrasound scans instead of just the standard single scan), is a possible limitation of the above study on foetal growth trajectory^1^. Indeed, when testing the hypothesis that frequent ultrasound scans during gestation would reduce the rate of adverse birth outcomes, Newnham et al.^2^ instead noticed that the babies in the “intensive imaging” group had lower birth weights than the control group, suggesting that the multiple ultrasound scans may have affected foetal growth. However, by 1 year of age, there were no longer any anthropomorphic differences between groups. Likewise, Forward et al.^3^ found no significant difference in AL, corneal curvature, lens thickness, or rates of myopia at age 20 between the “intensive imaging” and control groups. Thus, frequent ultrasound scans during gestation do not affect myopia rates.

### Sleep duration and quality

Studies exploring association between sleep duration and myopia have reported conflicting findings.^4-12^ However, many of these studies are limited by their cross-sectional design, when sleep duration was only obtained retrospectively or at the time of refractive error measurement, and thus the shorter sleep duration in myopes may reflect a tendency for myopes to sleep less, rather than a causal link. Longitudinal prospective studies on this issue have failed to find a significant link between sleep duration and myopia,^9,12,13^ indicating that this relationship, if any, is unlikely to be clinically important.

Sleep can be quantified not only by the duration but also by quality. Some studies have noted that individuals with myopia tend to report poorer sleep quality than those without myopia.^7,14^ However, the cross-sectional nature of these studies does not permit teasing out whether the sleep disturbances occur prior to the development of myopia. Using prospectively-collected data from 5- to 17-year-old children, a “sleep behavior trajectory” was modelled based on parent-reported information on the sleep quality of 1,194 Gen2 participants from the Raine Study. Participants were identified as having “minimal”, “declining”, or “persistent” sleep problems over the 13-year period.^15^ Importantly, sleep quality was captured in the years prior to and during myopia development and collected prior to the eye examination at 20 years old. No significant difference between groups in rates or severity of myopia, or in any of ocular biometric measures at age 20 were found,^16^ showing limited evidence of any effect of sleep quality on myopia development.

### Dietary Vitamin A

Vitamin A deficiency is known to cause night blindness and xerophthalmia, along with a myriad of systemic morbidities, most notably in lesser resourced countries.^17^ Given that retinoic acid, a metabolite of Vitamin A, has been shown to regulate eye growth in animal models,^18-20^ possible links between reduced Vitamin A intake and longer eyes, and thus myopia have been suggested. The Raine Study determined Vitamin A intake using the Food Frequency Questionnaire at the Gen2 14-, 17-, and 20-year follow-ups and found no association between Vitamin A intake and myopia or its related measures.^21^

**References**

1. Dyer KIC, Sanfilippo PG, White SW, et al. Associations Between Fetal Growth Trajectories and the Development of Myopia by 20 Years of Age. *Invest Ophthalmol Vis Sci.* 2020;61(14):26.

2. Newnham JP, Evans SF, Michael CA, Stanley FJ, Landau LI. Effects of frequent ultrasound during pregnancy: a randomised controlled trial. *Lancet.* 1993;342(8876):887-891.

3. Forward H, Yazar S, Hewitt AW, et al. Multiple prenatal ultrasound scans and ocular development: 20-year follow-up of a randomized controlled trial. *Ultrasound Obstet Gynecol.* 2014;44(2):166-170.

4. Jee D, Morgan IG, Kim EC. Inverse relationship between sleep duration and myopia. *Acta ophthalmologica.* 2016;94(3):e204-210.

5. You QS, Wu LJ, Duan JL, et al. Factors associated with myopia in school children in China: the Beijing childhood eye study. *PloS one.* 2012;7(12):e52668.

6. Xu C, Pan C, Zhao C, et al. Prevalence and risk factors for myopia in older adult east Chinese population. *BMC Ophthalmol.* 2017;17(1):191.

7. Zhou Z, Morgan IG, Chen Q, Jin L, He M, Congdon N. Disordered sleep and myopia risk among Chinese children. *PloS one.* 2015;10(3):e0121796.

8. Sensaki S, Sabanayagam C, Chua S, et al. Sleep Duration in Infants Was Not Associated With Myopia at 3 Years. *Asia Pac J Ophthalmol (Phila).* 2018;7(2):102-108.

9. Wei SF, Li SM, Liu L, et al. Sleep Duration, Bedtime, and Myopia Progression in a 4-Year Follow-up of Chinese Children: The Anyang Childhood Eye Study. *Invest Ophthalmol Vis Sci.* 2020;61(3):37.

10. Loman J, Quinn GE, Kamoun L, et al. Darkness and near work: myopia and its progression in third-year law students. *Ophthalmology.* 2002;109(5):1032-1038.

11. Pan CW, Liu JH, Wu RK, Zhong H, Li J. Disordered sleep and myopia among adolescents: a propensity score matching analysis. *Ophthalmic Epidemiol.* 2019:1-6.

12. Rayapoulle A, Gronfier C, Forhan A, Heude B, Charles MA, Plancoulaine S. Longitudinal association between sleep features and refractive errors in preschoolers from the EDEN birth-cohort. *Sci Rep.* 2021;11(1):9044.

13. Liu XN, Naduvilath TJ, Wang J, et al. Sleeping late is a risk factor for myopia development amongst school-aged children in China. *Sci Rep.* 2020;10(1):17194.

14. Ayaki M, Torii H, Tsubota K, Negishi K. Decreased sleep quality in high myopia children. *Sci Rep.* 2016;6:33902.

15. McVeigh JA, Smith A, Howie EK, et al. Developmental trajectories of sleep during childhood and adolescence are related to health in young adulthood. *Acta Paediatr.* 2021;110(8):2435-2444.

16. Stafford-Bell N, McVeigh J, Lingham G, et al. Associations of 12-year sleep behaviour trajectories from childhood to adolescence with myopia and ocular biometry during young adulthood. *Ophthalmic & physiological optics : the journal of the British College of Ophthalmic Opticians.* 2021.

17. Mayo-Wilson E, Imdad A, Herzer K, Yakoob MY, Bhutta ZA. Vitamin A supplements for preventing mortality, illness, and blindness in children aged under 5: systematic review and meta-analysis. *Bmj.* 2011;343:d5094.

18. Bitzer M, Feldkaemper M, Schaeffel F. Visually induced changes in components of the retinoic acid system in fundal layers of the chick. *Exp Eye Res.* 2000;70(1):97-106.

19. McFadden SA, Howlett MH, Mertz JR. Retinoic acid signals the direction of ocular elongation in the guinea pig eye. *Vision research.* 2004;44(7):643-653.

20. Troilo D, Nickla DL, Mertz JR, Summers Rada JA. Change in the synthesis rates of ocular retinoic acid and scleral glycosaminoglycan during experimentally altered eye growth in marmosets. *Invest Ophthalmol Vis Sci.* 2006;47(5):1768-1777.

21. Ng FJ, Mackey DA, O'Sullivan TA, Oddy WH, Yazar S. Is Dietary Vitamin A Associated with Myopia from Adolescence to Young Adulthood? *Translational vision science & technology.* 2020;9(6):29.
